# Incidence and Risk Factors of De Novo Postpartum Hypertension: Understanding Links to Long-Term Cardiovascular Risk

**DOI:** 10.1016/j.jacadv.2025.101756

**Published:** 2025-05-02

**Authors:** Ukachi N. Emeruwa, Ana Çapi, Marni B. Jacobs, Louise C. Laurent, Natalie A. Bello, Cynthia Gyamfi-Bannerman

**Affiliations:** a Division of Maternal-Fetal Medicine, Department of Obstetrics, Gynecology, and Reproductive Sciences, University of California-San Diego School of Medicine, UC San Diego Health, La Jolla, California, USA; b Department of Obstetrics and Gynecology, Icahn School of Medicine at Mount Sinai, New York, New York, USA; c Department of Obstetrics, Gynecology, and Reproductive Sciences, University of California San Diego School of Medicine, UC San Diego Health, La Jolla, California, USA; d Department of Cardiology, Smidt Heart Institute, Cedars-Sinai Medical Center, Los Angeles, California, USA

**Keywords:** cardiovascular disease risk, de novo postpartum hypertension, gestational diabetes mellitus, maternal morbidity, postpartum hypertension, pregnancy-related hypertension

## Abstract

**Background:**

Individuals developing hypertensive disorders of pregnancy face a 2- to 5-fold risk of long-term cardiovascular disease. Limited data exist on de novo postpartum hypertension (dnPPHTN), where those normotensive during pregnancy develop hypertension immediately postpartum. Under-recognition of dnPPHTN can lead to severe morbidity due to delayed or absent treatment and missed opportunities for mitigating long-term cardiovascular disease risk.

**Objectives:**

The aim of the study was to estimate the incidence of dnPPHTN and identify demographic and clinical risk factors for its development.

**Methods:**

This retrospective cohort study analyzed 506 postpartum individuals delivering at a tertiary care institution over 1 month. Participants were classified as: 1) normotensive; or 2) dnPPHTN, defined as systolic blood pressure (BP) ≥140 mm Hg and/or diastolic BP ≥90 mm Hg on at least 2 occasions up to 6 weeks postpartum after a normotensive pregnancy. We excluded those with prepregnancy or antepartum hypertensive disorders. Demographic and clinical characteristics were compared using adjusted logistic regression models.

**Results:**

Of 389 included participants, 35 (9.0%) developed dnPPHTN. Of these, 5.7% had pregestational diabetes compared to 0.6% of normotensive individuals (*P* = 0.042; adjusted OR: 11.3; 95% CI: 1.8-73.1). Early prenatal diastolic BP was higher in the dnPPHTN group (72.2 vs 68.4 mm Hg, *P* = 0.008), though this difference did not persist after adjustment. Medication-dependent gestational diabetes mellitus (ie, A2GDM) was associated with dnPPHTN (adjusted OR: 6.1; 95% CI: 1.2-30.1).

**Conclusions:**

Pregestational diabetes and A2GDM are associated with dnPPHTN. Closer follow-up for BP monitoring postpartum and more urgent transitions of care for ongoing medical management may reduce long-term cardiovascular risk.

Hypertensive disorders of pregnancy (HDP), which include chronic (prepregnancy) hypertension, gestational hypertension, pre-eclampsia, and hemolysis, elevated liver enzymes, low platelets (HELLP) syndrome, rank among the top 3 causes of maternal morbidity and mortality worldwide.^1^^,^^2^ They complicate every 1 in 7 pregnancies,^1^^,^^3^ are associated with 3 to 8 times higher odds of acute cardiovascular and cerebrovascular complications (eg, myocardial infarction, peripartum cardiomyopathy, stroke, and so on) in pregnancy compared to normotensive pregnancies,^4^ and lead to over 70,000 maternal deaths.^5^ Recent data indicate that 70% of hypertension-related maternal deaths occur after delivery.^6^ Beyond the pregnancy-associated morbidity and mortality, long-term adverse outcomes of HDP are well-documented. Birthing people who develop HDP face a 2- to 5-fold increased risk of long-term cardiovascular disease.^4^^,^^7^

There is a limited knowledge about the clinical entity known as de novo postpartum hypertension (dnPPHTN), in which people who are normotensive throughout pregnancy and delivery subsequently go on to develop a hypertensive disorder in the postpartum period. DnPPHTN is a relatively new and variably defined entity, often used to describe new-onset hypertension in the postpartum period regardless of the presence of additional markers for pre-eclampsia. The focus on elevated blood pressure (BP)—with or without proteinuria or other signs of pre-eclampsia-related end-organ compromise—reflects the clinical urgency associated with cerebrovascular and cardiovascular complications that may occur even at moderately elevated pressures. Prior studies have demonstrated increased risk of peripartum hemorrhagic stroke at systolic pressures ≥160 mm Hg, even in the setting of normal or only mildly elevated diastolic pressures.^8^^,^^9^ Proteinuria, while relevant as 1 diagnostic indicator for pre-eclampsia, is not consistently assessed in the postpartum setting due to contamination from lochia and limited implications for immediate management, among other possible considerations.

Given that one- to two-thirds of patients with postpartum pre-eclampsia were normotensive antepartum,^10^ the lack of knowledge about dnPPHTN results in a highly vulnerable population. Prenatal care protocols include at least once weekly BP evaluation for all patients in the month before estimated delivery; conversely, postpartum protocols for normotensive antepartum patients have no BP monitoring recommendations until 6 weeks postpartum.^11^ This evaluation comes after 75% of dnPPHTN cases (90% of severe dnPPHTN cases) have already occurred.^12^^,^^13^ Therefore, in the absence of research identifying risk factors and surveillance strategies, patients with de novo postpartum hypertensive disorders are frequently unrecognized until they present with hypertension-associated severe maternal morbidities, such as pulmonary edema and stroke. Even for patients with more indolent courses of dnPPHTN, which pose long-term health risks, there remains a missed opportunity for timely transitions of care to primary care and cardiology providers for ongoing management to facilitate long-term reduction in cardiovascular disease morbidity.

Our analysis aimed to fill this knowledge gap by estimating the incidence of dnPPHTN and identifying associated demographic and clinical characteristics in this high-risk population during pregnancy. The results of this study may provide useful information for risk stratification that will be helpful in guiding surveillance, management, and preventive strategies that may ultimately reduce short- and long-term morbidity associated with postpartum hypertensive disorders.

## Methods

### Study design

We conducted a retrospective cohort study of obstetric patients delivering at 20 weeks of gestation or greater at NewYork-Presbyterian Morgan Stanley Children's Hospital during the month of December 2019. This is a high-volume tertiary care hospital located in northern Manhattan and affiliated with Columbia University Irving Medical Center. This study was approved by the Columbia University Irving Medical Center Institutional Review Board with a waiver of informed consent. The findings in this study follow the Strengthening the Reporting of Observational Studies in Epidemiology guidelines.

### Study population and outcomes

Demographic and clinical information were abstracted from the electronic medical record for evaluation of outcomes. Patients with chronic hypertension, gestational hypertension, pre-eclampsia, or HELLP syndrome diagnosed before delivery, and patients without postpartum follow-up data were excluded from our study. Included patients were classified into 2 groups: normotensive or having dnPPHTN. Normotensive was defined as systolic blood pressure (SBP) <140 mm Hg and diastolic blood pressure (DBP) <90 mm Hg and included patients with erroneous or nonsustained mildly elevated BP measurements. The primary outcome, dnPPHTN, was defined in accordance with the American College of Obstetricians and Gynecologists guidelines as SBP ≥140 mm Hg and/or DBP ≥90 mm Hg, following the delivery of a normotensive pregnancy, confirmed on at least 2 separate measurements ≥4 hours apart within 6 weeks postdelivery. Due to inconsistent assessment of proteinuria and laboratory markers in routine postpartum clinical practice, we did not distinguish between dnPPHTN and pre-eclampsia; all cases meeting the BP criteria were categorized as dnPPHTN. Outcome assessment included review of BPs after delivery before hospital discharge, as well as identification of office visits, emergency department or obstetric triage visits, or hospital readmissions within 6 weeks postpartum.

### Covariates

Detailed demographic, obstetric, and clinical data were collected for evaluation of covariates. Demographic data included age, self-reported race and ethnicity (given known racial and ethnic disparities in HDP and maternal morbidity), and insurance payer classification (public vs private). Obstetric data included gravidity; parity, delivery interval >10 years, mode of delivery (vaginal vs cesarean), spontaneous pregnancy vs pregnancy resulting from in vitro fertilization, prepregnancy body mass index (BMI), BMI at delivery, systolic and diastolic BP at intake prenatal visit (where recorded before 20 weeks of gestation, ie, “booking BP”), history of pre-eclampsia in a prior pregnancy, and diagnosis of gestational diabetes in index pregnancy. Fetal characteristics associated with HDP were also abstracted, including gestational age at delivery, plurality (ie, singleton vs multifetal pregnancy), and fetal growth restriction at the time of delivery. Clinical data included maternal comorbidities associated with HDP, specifically pregestational diabetes, autoimmune disease, renal disease, antiphospholipid antibody syndrome, smoking status, and alcohol use. All data were adjudicated by 2 clinical investigators (U.N.E. and A.C.).

### Statistical analysis

Demographic, obstetric, and clinical data were compared for participants classified as normotensive vs those developing dnPPHTN. Continuous variables were assessed for normality graphically. Non-normally distributed data were compared using the Wilcoxon rank-sum test and are displayed as median with 25th and 75th percentiles (Q1-Q3); normally distributed data were compared using the Student *t*-test and are displayed as mean ± SD. Pearson chi-square test or Fisher exact test was used to compare categorical variables where appropriate. We fit unadjusted and adjusted logistic regression models for these characteristics including confounders of statistical significance as determined by *P* value < 0.20 in between group comparisons. As the goal of this study was to identify clinical characteristics potentially associated with dnPPHTN, we selected an explanatory modeling approach prioritizing interpretability over prediction. Given the sample size and study design, predictive modeling techniques—which typically require larger datasets to accommodate a broader range of covariates—were not appropriate for this analysis. In the multivariable analysis, 2 separate models were fit due to collinearity between pregestational diabetes (model A) and medication-dependent gestational diabetes mellitus (A2GDM) (model B). Pregestational diabetes and A2GDM were modeled as separate covariates given their distinct definitions and pathophysiologic mechanisms. Pregestational diabetes is a well-established risk factor for HDP, while the role of A2GDM in the development of postpartum hypertension remains less well characterized. Distinguishing their independent contributions may yield clinically meaningful insights into postpartum hypertension pathophysiology. ORs and adjusted ORs (aORs) with 95% CI are presented as measures of association. Race was included in each model given its established role as a social and structural confounder and its statistically significant difference between groups but should not be misinterpreted as a biologically or clinically actionable risk factor. All analyses were conducted using Stata/IC Version 16.0 (StataCorp, 2019). A 2-sided *P* value <0.05 was considered statistically significant.

## Results

**Figure 1:**
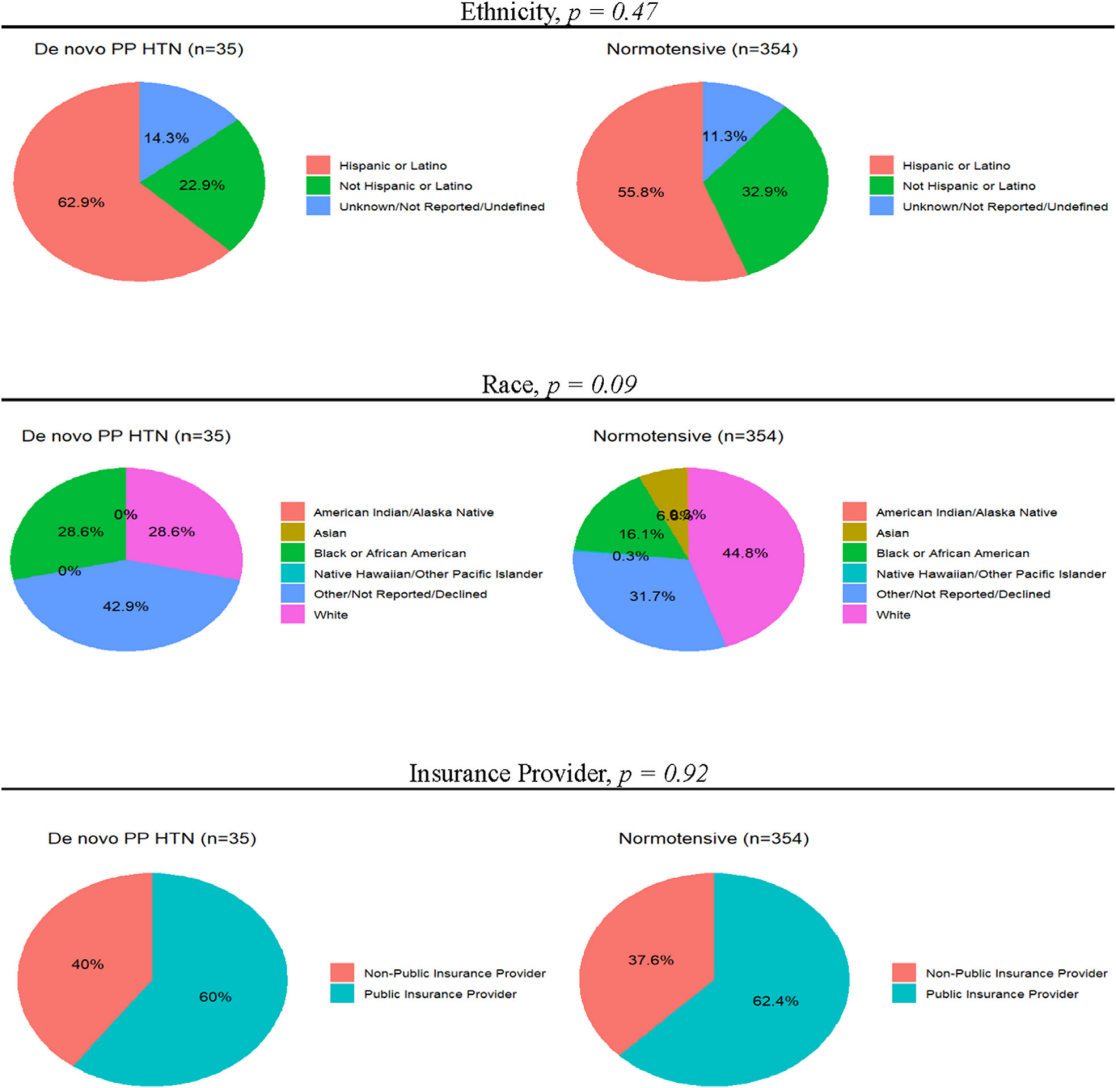
Sociodemographic Makeup of Cohort The ethnic, racial, and insurance provider makeup of those who remained normotensive and those who developed de novo postpartum hypertension (dnPPHTN) are shown above. *P* values provided above were derived from comparisons using Pearson chi-square test or Fisher exact test where appropriate. HTN = hypertension; PP = postpartum.

A total of 506 deliveries during the study period were reviewed for eligibility. Of these, 112 participants were excluded due to an antenatal diagnosis of hypertension or HDP. Five patients were excluded due to lack of postpartum follow-up data. Of 389 participants who were normotensive through delivery, 35 (9.0%) developed dnPPHTN. The sociodemographic makeup of those who remained normotensive postpartum and those who developed dnPPHTN is shown in Figure 1. Overall mean maternal age was 31 ± 6 years. The median pregravid BMI for the cohort was 25.5 kg/m^2^ (Q1-Q3: 22.6-29.6 kg/m^2^) with 111 (28.5%) meeting criteria for obesity (BMI ≥30 kg/m^2^) and 59 (15.2%) meeting criteria for Class II+ obesity (BMI ≥35 kg/m^2^). Pregravid BMI and rates of obesity were not significantly different between those who remained normotensive postpartum and those who developed dnPPHTN. Demographic and clinical characteristics were similar between those who remained normotensive postpartum and those who developed dnPPHTN (Table 1).

**Table 1:** Demographic and Clinical Characteristics

	De Novo PP HTN (n = 35)	Normotensive (n = 354)	*P* Value
Demographic characteristics			
Maternal age, y	29.8 ± 7.0	30.6 ± 6.5	0.46
Gravidity	3 (1.0, 4.5)	2.0 (1.0, 3.0)	0.44
Parity	0.0 (0.0, 2.0)	1.0 (0.0, 1.0)	0.35
Ethnicity			0.42
Hispanic or Latino	22 (62.9)	197 (55.8)	
Not Hispanic or Latino	8 (22.9)	116 (32.9)	
Unknown/not reported/declined	5 (14.3)	40 (11.3)	
Race			0.091
American Indian/Alaska Native	0 (0.0)	1 (0.3)	
Asian	0 (0.0)	24 (6.8)	
Black or African American	10 (28.6)	57 (16.1)	
Native Hawaiian/Other Pacific Islander	0 (0.0)	1 (0.3)	
White	10 (28.6)	158 (44.6)	
Other/not reported/declined	15 (42.9)	113 (31.9)	
BMI, kg/m^2^			
Pregravid	26.3 (24.1, 29.4)	25.1 (22.5, 29.5)	0.40
At delivery	30.9 (27.8, 33.6)	29.9 (26.9, 34.0)	0.26
Gestational age at delivery, wk	39.0 (38.6, 39.9)	39.0 (38.1, 39.7)	0.93
Clinical characteristics			
Antiphospholipid antibody syndrome	0 (0.0)	2 (0.6)	>0.99
Autoimmune disease	2 (5.7)	15 (4.3)	0.66
Booking blood pressure, mm Hg			
Systolic	111.7 ± 12.2	107.8 ± 10.0	0.058
Diastolic	72.2 ± 7.0	68.4 ± 7.3	0.008
Delivery interval >10 y (n = 15, n = 206)	2 (13.3)	15 (7.3)	0.32
Fetal growth restriction at delivery	4 (11.4)	18 (5.2)	0.13
History of pre-eclampsia in a prior pregnancy	1 (6.7)	11 (5.3)	0.58
Multiples	0 (0.0)	13 (3.7)	0.62
Obesity			
Class I+	8 (22.9)	103 (29.1)	0.56
Class II+	3 (8.6)	56 (15.8)	0.33
Total weight gain, lbs	28.1 (18.6, 36.5)	24.1 (15.4, 31.9)	0.12
Pregestational diabetes	2 (5.7)	2 (0.6)	0.042
Gestational diabetes (n = 30, n = 327)			
Any (A1 or A2)	4 (13.3)	27 (8.3)	0.31
Medication-dependent (A2)	2 (6.7)	6 (1.8)	0.14
Renal disease	1 (2.9)	2 (0.6)	0.25

Values are mean ± SD, median (Q1,Q3), or n (%). Demographic and clinical characteristics were compared between those who remained normotensive and those who developed de novo postpartum hypertension (dnPPHTN). Continuous variables were assessed for normality graphically. Non-normally distributed data were compared using the Wilcoxon rank-sum test; normally distributed data were compared using Student t-test. Pearson chi-square test or Fisher exact test was used to compare categorical variables where appropriate.

For variables with incomplete data, the number of participants with available data in the dnPPHTN and normotensive groups, respectively, is shown in parentheses.

BMI = body mass index; HTN = hypertension; PP = postpartum.

Though both groups had booking BPs (ie, intake prenatal BPs recorded before 20 weeks of gestation) in the normal range, participants with dnPPHTN had higher diastolic booking BPs compared to normotensive participants. The mean DBP before 20 weeks gestation was 72.2 ± 7.0 mm Hg vs 68.4 ± 7.3 mm Hg, respectively (*P* = 0.008). The difference in the mean SBP did not reach statistical significance (systolic booking BP 111.7 ± 12.2 mm Hg for participants with dnPPHTN vs 107.8 ± 10.0 mm Hg for normotensive patients, *P* = 0.058). Unadjusted and adjusted logistic regression models are shown in Tables 2 and 3. The difference in diastolic booking BP did not persist after controlling for confounders (specifically race, fetal growth restriction at the time of delivery, booking SBP and DBP, total weight gain, and pregestational diabetes), though a marginal association remained in the model with A2GDM. Rates of pregestational diabetes were higher among participants who developed dnPPHTN (5.7%) compared to those who remained normotensive (0.6%, *P* = 0.042). This association persisted in adjusted analyses (aOR: 11.3; 95% CI: 1.8-73.1). After controlling for confounders, A2GDM was associated with dnPPHTN (aOR: 6.1; 95% CI: 1.2-30.1).

**Table 2:** Univariable and Multivariable Logistic Regression of Characteristics Associated With De Novo Postpartum Hypertension (Model A: Pregestational Diabetes)

	Unadjusted OR (95% CI)	Adjusted OR (95% CI)
Pregestational diabetes	10.67 (1.45-78.20)	11.31 (1.76-73.11)
Fetal growth restriction at delivery	2.37 (0.76-7.45)	2.72 (0.81-9.06)
Booking blood pressure, mm Hg		
Systolic	1.04 (1.00-1.08)	1.00 (0.95-1.05)
Diastolic	1.07 (1.02-1.13)	1.06 (0.99-1.14)
Total weight gain, lbs	1.01 (0.99-1.04)	1.00 (0.99-1.01)
Race	1.18 (1.00-1.39)	1.23 (1.02-1.49)

Multivariable logistic regression models were fit to perform adjusted analyses, including confounders of statistical significance (listed in the table above) as determined by a *P* value <0.20 in between-group comparisons. Race was included given its established role as a social and structural confounder and its statistically significant difference between groups but should not be misinterpreted as a biologically or clinically actionable risk factor. This model includes pregestational diabetes and other associated characteristics (n = 291; 29 cases of de novo postpartum hypertension; 6 degrees of freedom). Unadjusted and adjusted ORs are presented as measures of association.

**Table 3:** Univariable and Multivariable Logistic Regression of Characteristics Associated With De Novo Postpartum Hypertension (Model B: Gestational Diabetes, A2)

	Unadjusted OR (95% CI)	Adjusted OR (95% CI)
Gestational diabetes, A2	10.67 (1.45-78.20)	6.08 (1.22-30.11)
Fetal growth restriction at delivery	2.37 (0.76-7.45)	3.09 (0.89-10.66)
Booking blood pressure, mm Hg		
Systolic	1.04 (1.00-1.08)	1.00 (0.95-1.05)
Diastolic	1.07 (1.02-1.13)	1.08 (1.01-1.16)
Total weight gain, lbs	1.01 (0.99-1.04)	1.00 (0.99-1.02)
Race	1.18 (1.00-1.39)	1.21 (1.00-1.47)

Multivariable logistic regression models were fit to perform adjusted analyses, including confounders of statistical significance (listed in the table above) as determined by a *P* value <0.20 in between-group comparisons. Race was included given its established role as a social and structural confounder and its statistically significant difference between groups but should not be misinterpreted as a biologically or clinically actionable risk factor. This model includes A2 gestational diabetes and other associated characteristics (n = 291; 29 cases of de novo postpartum hypertension; 6 degrees of freedom). Unadjusted and adjusted ORs are presented as measures of association.

## Discussion

In this retrospective cohort study, dnPPHTN was associated with higher rates of pregestational diabetes mellitus and A2GDM compared to patients who remained normotensive through the postpartum period. (Central Illustration) An apparent difference in early prenatal BPs between the 2 groups in our population did not persist after controlling for confounders.

**Central Illustration:**
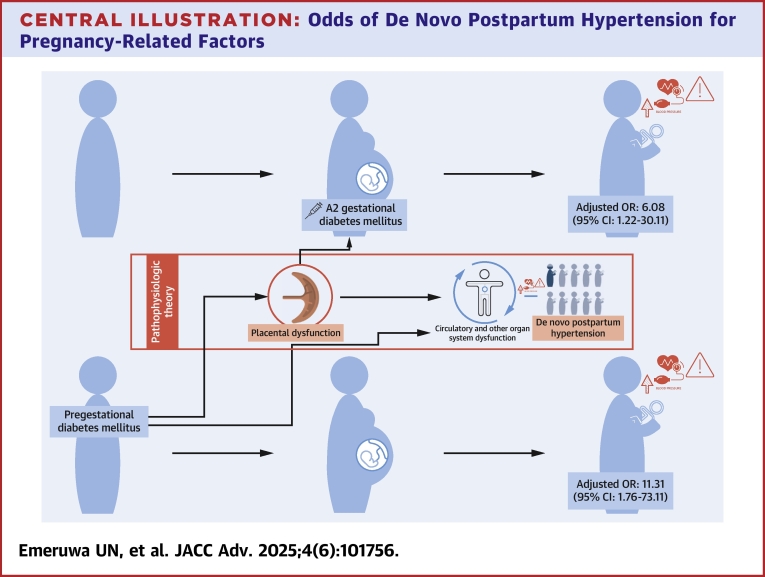
Odds of De Novo Postpartum Hypertension for Pregnancy-Related Factors De novo postpartum hypertension occurred in 9% of the cohort. Pregestational diabetes mellitus (aOR: 11.31; 95% CI: 1.76-73.11) and medication-dependent (A2) gestational diabetes mellitus (aOR: 6.08; 95% CI: 1.22-30.11) were associated with increased odds of de novo postpartum hypertension. We propose that both conditions are linked to de novo postpartum hypertension through their relationship to placental dysfunction. Pregestational diabetes, a well-established risk factor for antenatal hypertensive disorders, can cause direct placental vascular damage and/or impair the maternal vascular system's ability to adapt to pregnancy-induced changes mediated by placental dysfunction. Gestational diabetes, like hypertensive disorders of pregnancy, represents a pregnancy-specific manifestation of placental dysfunction, particularly affecting endocrine regulation. aOR = adjusted OR.

The incidence of dnPPHTN estimated in our study is 9%, aligning with other published data.^10^^,^^12^, ^13^, ^14^ Until 2022, the limited available studies on risk factors for dnPPHTN focused on populations of patients readmitted with new-onset hypertension and related complications.^15^^,^^16^ These investigations have been critical in identifying obstetric patients at high risk for hypertensive complications in the immediate postpartum period but are limited in their ability to characterize patients with milder presentations of postpartum hypertension who may still be at increased risk for long-term cardiovascular sequelae. This study fills a critical gap by examining a broader, under-monitored, at-risk postpartum population, offering valuable insights into the under-recognized condition of dnPPHTN and its potential long-term impact on maternal health. Notably, this study directly informed the postpartum risk stratification algorithm used in the LAPP (Lasix for the prevention of de novo postpartum hypertension) randomized clinical trial.^13^ In particular, the observed association between A2GDM and dnPPHTN served as a rationale for the inclusion of this clinical diagnosis into the LAPP Trial's “high-risk” eligibility criteria.

A 2023 retrospective study of 3,925 patients delivering at a safety-net hospital offered descriptive insights into dnPPHTN by performing chart reviews for all comers at risk of dnPPHTN but did not perform formal statistical testing or adjust for potential confounders.^12^ Our study builds on this by attempting to establish associations that may generate hypotheses for causal relationships or mechanistic insights in a similar cohort of at-risk individuals.

One finding of note that appears to have benefitted from such analysis is the newly identified association between gestational diabetes and dnPPHTN. Similar to the findings in the aforementioned Parker et al study, in an unadjusted analysis, there appears to be no association. In our study, controlling for potential confounders and stratifying by gestational diabetes severity reveals a statistically significant link. Gestational diabetes, like HDP, is an obstetric disorder induced by placental dysfunction; thus, it is biologically plausible that there may be a pathophysiologic link between the two. This finding is particularly important because patients with A2GDM are already at increased risk for type 2 diabetes mellitus. The association with postpartum hypertension and long-term cardiovascular risk indicates a broader susceptibility to metabolic syndrome in these patients. This presents an opportunity for early intervention and targeted prevention in a young, at-risk population before the onset of chronic disease. Enhanced BP surveillance and timely transitions of care would address evolving comorbidities at their earliest stages in order to mitigate long-term cardiovascular risks. Although our study is observational, it lays important groundwork for future research to develop evidence-based guidelines and management strategies to reduce morbidity in patients with A2GDM and prevent metabolic syndrome.

Pregestational diabetes is a well-established risk factor for antenatal pre-eclampsia due to the baseline vascular and renal compromise and susceptibility it confers. Our study also found an association between pregestational diabetes and dnPPHTN, which has been described in studies of patients readmitted for dnPPHTN, indicating that pregnancy-related risks persist after delivery. Enhanced BP protocols for patients with pregestational diabetes or A2GDM during the postpartum period could be incorporated into clinical guidelines to reduce the risk of undiagnosed pregnancy-related hypertension by improving early detection and intervention. Coupled with timely referrals to cardiology and primary care, these protocols could help decrease the likelihood of long-term cardiovascular complications.

Our study's robustness stems from its execution at a high-volume tertiary care center, which allowed for a comprehensive review and analysis of nearly 600 patients, of whom almost 400 were at risk for the primary outcome within a single month. Through meticulous examination of outpatient and various hospital-based visit settings, our research identifies more cases of dnPPHTN than those previously identified solely based on readmission data. Furthermore, our commitment to careful adjudication of patient demographic and clinical data ensured high data completeness, enabling adjusted analyses that provide a more nuanced understanding of the factors associated with dnPPHTN. These associations serve as a steppingstone for future research aimed at establishing causal links and developing targeted interventions. As such, our findings contribute to a deeper understanding of dnPPHTN and pave the way for advancements in postpartum care that could significantly improve both short- and long-term maternal health outcomes.

### Study limitations

There are a few limitations to acknowledge. First, the single-center design reduces study generalizability, though our cohort's demographic diversity reflects the population diversity seen in many urban centers, which may make these findings applicable to a range of clinical settings. Second, the limited data collection timeframe limits statistical power, particularly regarding the identification of other potential characteristics and conditions associated with dnPPHTN. Additionally, while missing data were minimal and not expected to significantly bias the results, we acknowledge that the absence of imputation methods or sensitivity analyses is a limitation. Finally, the diagnosis of dnPPHTN relied on chart review of medical setting touchpoints. In accordance with the current standard of care, aside from delivery hospitalization vital sign monitoring, BP measurements are limited to 1 or 2 outpatient visits in the 6-week postpartum period. This method of ascertainment, along with the retrospective nature of the study, may lead to misclassification bias and hampers our ability to accurately estimate the true incidence of dnPPHTN. More frequent or home BP monitoring would be necessary for precise estimations of dnPPHTN incidence.

## Conclusions

Our study found a 9% rate of dnPPHTN in the 6 weeks following delivery in our cohort. Pregestational diabetes and A2GDM are associated with the development of dnPPHTN in this population. Further exploration of these and other potential risk factors for dnPPHTN may identify patients who may benefit from closer follow-up for BP monitoring in the postpartum period and more urgent transitions of care for long-term cardiovascular disease risk reduction.

Perspectives **COMPETENCY IN MEDICAL KNOWLEDGE AND SYSTEMS-BASED PRACTICE:** DnPPHTN is an under-recognized yet increasingly prevalent contributor to postpartum cardiovascular morbidity, affecting approximately 1 in 11 patients who were normotensive during pregnancy. This study identifies early clinical factors—particularly A2GDM and pregestational diabetes—as being associated with increased odds of dnPPHTN, suggesting potential shared mechanisms with other forms of placental dysfunction and hypertensive disorders of pregnancy. These findings offer actionable insights for prenatal risk stratification and tailored postpartum BP monitoring, particularly for individuals without hypertensive disease during pregnancy. Given the growing emphasis on extending cardiovascular risk prevention into the postpartum period, these data provide a strong clinical foundation to improve systems-level care delivery and outcomes for birthing people.

**TRANSLATIONAL OUTLOOK:** Current postpartum protocols often delay or omit BP monitoring for patients who were normotensive during pregnancy, despite growing evidence that a substantial proportion of postpartum hypertensive events occur in this group. These findings support earlier postpartum surveillance and referral for patients with prenatal metabolic risk factors. Future research should focus on validating early postpartum predictors in larger, diverse populations and evaluating risk-based monitoring strategies that balance sensitivity with implementation feasibility. This study directly informed the postpartum risk stratification algorithm used in the LAPP Trial—a randomized controlled trial incorporating remote BP monitoring in the evaluation of diuretic therapy for the prevention of dnPPHTN—highlighting the translational potential of identifying early risk factors. Prospective and implementation-focused studies are needed to determine whether integrated monitoring strategies and targeted interventions can reduce postpartum morbidity and long-term cardiovascular risk in this population.

## Funding support and author disclosures

Dr Emeruwa's time was supported by the NICHD K12 (HD001259-25) grant and Robert A. Winn Career Development Award. The authors have reported that they have no relationships relevant to the contents of this paper to disclose.
